# Building the primary cilium membrane

**DOI:** 10.1186/2046-2530-1-S1-O15

**Published:** 2012-11-16

**Authors:** CJ Westlake, J Rahajeng, Q Lu, RH Scheller, S Caplan, PK Jackson

**Affiliations:** 1 NIH-NCI, USA; 2 University of Nebraska Medical Center, USA; 3 Genentech, USA

## 

Ciliogenesis involves coordinated assembly of a microtubule-based axoneme from the mother centriole and vesicular membrane transport and fusion forming a ciliary membrane around the developing axoneme. We, and others have reported that a Rab11-Rab8 cascade functions in ciliogenesis. Using live high-resolution fluorescence microscopy imaging we show that ciliary membrane assembly proceeds following Rabin8 (a Rab8 activator) binding to Rab11 membranes. Rabin8 transport via Rab11 vesicles to the centrosome is observed resulting in localized activation of Rab8 and leads to initiation of ciliary membrane assembly. Using proteomics approaches, we have discovered that Rabin8 binds to the TRAPPII tethering complex and find that this interaction is important for Rabin8 centrosomal targeting during ciliogenesis. Our work suggests that Rabin8 membrane transport is a highly regulated process controlled by serum-dependent and serum-independent signaling. Interestingly, following ciliogenesis Rabin8 centrosomal localization is lost resulting in reduced Rab8 activation at the ciliary membrane. This finding along with a previous report describing Rabin8 association with Bardet-Biedl syndrome (BBS) proteins has led us to hypothesize that regulation of centrosomal Rabin8 levels is important for establishing the length of primary cilium, an important factor in ciliary signaling. Finally, we describe the discovery of additional factors associated with the Rab11-Rab8 trafficking pathway that function in organizing membrane structure during ciliogenesis.

